# Nasal carriage of methicillin resistant *Staphylococcus aureus* among health care workers at a tertiary care hospital in Western Nepal

**DOI:** 10.1186/s13756-015-0082-3

**Published:** 2015-10-09

**Authors:** Rita Khanal, Prakash Sah, Pramila Lamichhane, Apsana Lamsal, Sweety Upadhaya, Vijay Kumar Pahwa

**Affiliations:** Department of Microbiology, Universal College of Medical Sciences and Teaching Hospital, Bhairahawa, Nepal

**Keywords:** MRSA, Healthcare workers, Nasal carriage, Nepal

## Abstract

**Background:**

*Staphylococcus aureus* is a frequent cause of infections in both the community and hospital. Methicillin-resistant *Staphylococcus aureus* continues to be an important nosocomial pathogen and infections are often difficult to manage due to its resistance to multiple antibiotics. Healthcare workers are important source of nosocomial transmission of MRSA. This study aimed to determine the nasal carriage rate of *S. aureus* and MRSA among healthcare workers at Universal College of Medical Sciences and Teaching Hospital, Nepal and to determine antibiotic susceptibility pattern of the isolates.

**Methods:**

A cross-sectional study involving 204 healthcare workers was conducted. Nasal swabs were collected and cultured on Mannitol salt agar. Mannitol fermenting colonies which were gram positive cocci, catalase positive and coagulase positive were identified as *S. aureus*. Antibiotic susceptibility test was performed by modified Kirby-Bauer disc diffusion method. Methicillin resistance was detected using cefoxitin disc diffusion method.

**Results:**

Of 204 healthcare workers, 32 (15.7 %) were nasal carriers of *S. aureus* and among them 7 (21.9 %) were carrier of MRSA. Overall nasal carriage rate of MRSA was 3.4 % (7/204). Highest MRSA nasal carriage rate of 7.8 % (4/51) was found among nurses. Healthcare workers of both surgical wards and operating room accounted for 28.6 % (2/7) of MRSA carriers each. Among MRSA isolates inducible clindamycin resistance was observed in 66.7 % (2/3) of erythromycin resistant isolates.

**Conclusions:**

High nasal carriage of *S. aureus* and MRSA among healthcare workers (especially in surgery ward and operating room) necessitates improved infection control measures to be employed to control MRSA transmission in our setting.

## Background

*Staphylococcus aureus* is a frequent cause of infections in both the community and hospital. The Methicillin-resistant *S. aureus* (MRSA) has emerged as one of the commonest causes of hospital acquired infection and continues to remain an important factor contributing to failure of management [1]. MRSA strains are not only a problem in hospital as distinct strains have emerged in community too, which are referred to as Community acquired MRSA (CA-MRSA). CA-MRSA strains have spread in community settings and have also entered healthcare facilities [2].

Healthcare workers (HCWs) who are at interface between the hospital and the community may serve as agents of cross contamination of Hospital acquired MRSA (HA-MRSA) and CA-MRSA [3]. HCWs are the source of nosocomial transmission of MRSA in developing countries [4, 5]. The average nasal carriage rate of *S. aureus* and MRSA among HCWs has been shown to be 23.7 and 4.6 % respectively [3]. Different studies from Nepal have showed nasal carriage rate of *S. aurues* among HCWs to be 20.37 – 43.8 % [6–9].

Identification of patients and HCWs in outbreak settings colonized with MRSA combined with hand hygiene and other precautions have been shown to be effective in reducing the transmission and controlling the spread of MRSA. This study was undertaken to investigate the nasal carriage rate of *S. aureus* and MRSA among HCWs at our hospitals and to determine antibiotic susceptibility pattern of the isolates.

## Methods

This cross sectional study was carried out at Universal College of Medical Sciences and Teaching Hospital, Bhairahawa, Nepal during the period of November – December 2013. The study was approved by institutional review committee of Universal College of Medical Sciences, Bhairahawa, Nepal. Informed consent was taken from all the participants. Nasal swabs from 204 HCWs were collected before commencement of duties. HCWs with history of upper respiratory tract infection, fever, recent nasal surgery, diabetes, immunocompromisation, use of nasal medications, or antimicrobial therapy were excluded. Nasal swabs were collected from anterior nares of the HCWs using sterile cotton swabs (moistened with normal saline). The swab was introduced 2–3 cm in the nasal cavity and rotated 4–5 times both clockwise and anticlockwise. The swabs were then immediately transported to the Microbiology laboratory for further processing. Specimens were inoculated onto Mannitol salt agar (MSA) and incubated at 37 °C for 48 h. Mannitol fermenting colonies that were yellow or golden yellow were selected and sub-cultured on Nutrient agar (NA). Colonies on NA were subjected to Gram’s staining, catalase test and coagulase test. Gram positive cocci that were catalase positive and coagulase positive were identified as *S. aureus* [10]. Antibiotic susceptibility testing of all isolates was performed by modified Kirby Bauer disc diffusion method as recommended by CLSI guidelines [11]. The antibiotics used in the study were amikacin (30 μg), ceftriaxone (30 μg), cefoxitin (30 μg), ciprofloxacin (5 μg), cloxacillin (30 μg), clindamycin (2 μg), cotrimoxazole (1.25/23.75 μg), erythromycin (15 μg), gentamycin (10 μg), penicillin (10Units), teicoplanin (30 μg), tetracycline (30 μg) and vancomycin (30 μg). Inducible clindamycin resistance was detected by D-test. Methicillin resistance was detected using cefoxitin disc diffusion test. [11] Data was analyzed using SPSS 17.0.

## Result

**Fig. 1:**
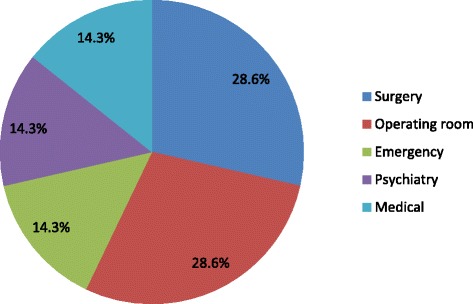
Ward wise distribution of MRSA

Of 204 HCWs, 32(15.7 %) were nasal carriers of *S. aureus* and among them 7(21.9 %) were carrier of MRSA. The overall nasal carriage rate of MRSA was 3.4 % (7/204). Nasal carriage among male and female HCWs were 19.4 % (21/108) and 11.5 % (11/96) respectively (*P* > 0.05). *S. aureus* carriage rate was highest among doctors 20.8 % (15/72) while MRSA carriage rate was highest among nurses 7.8 % (4/51) (Table 1). The highest rate of *S. aureus* carriers were found in HCWs of ophthalmology (60.0 %) and MRSA carriage rate of 40–50 % was found in surgery, operating room and emergency (Table 2). Among the MRSA found 28.6 % (2/7) were from HCWs of surgical wards and operating room each (Fig. 1).

**Table 1:** Prevalence of *S.aureus* and MRSA among healthcare workers

Healthcare workers	No of samples	*S. aureus* (%)	MRSA (%)
Doctor	72	15 (20.8)	1 (1.4)
Intern	36	6 (16.7)	1 (2.8)
Nurse	51	6 (11.8)	4 (7.8)
Attender	22	4 (18.2)	1 (4.5)
Laboratory personnel	16	1 (6.3)	0 (0)
Others	7	0 (0)	0 (0)
Total	204	32 (15.7)	7 (3.4)

**Table 2:** Distribution of *S.aureus* & MRSA among healthcare workers of different wards

Wards/Department	No of samples (*n* = 204)	*S. aureus* (%) (*n* = 32)	MRSA (%) (*n* = 7)
NICU	21	3 (14.3)	0 (0)
Surgery	27	5 (18.5)	2 (40.0)
Operating room	26	5 (19.2)	2 (40.0)
Orthopedics	17	1 (5.9)	0 (0)
Medical	23	3 (13.0)	1 (33.3)
Gynecology	16	2 (12.5)	0 (0)
Emergency	11	2 (18.2)	1 (50.0)
ICU	4	0 (0)	0 (0)
CCU	3	1 (33.3)	0 (0)
Dermatology	8	2 (25.0)	0 (0)
Ophthalmology	5	3 (60.0)	0 (0)
Psychiatry	9	3 (33.3)	1 (33.3)
ENT	4	0 (0)	0 (0)
Radiology	10	1 (10.0)	0 (0)
Others	20	1 (5.0)	0 (0)

**Fig. 2:**
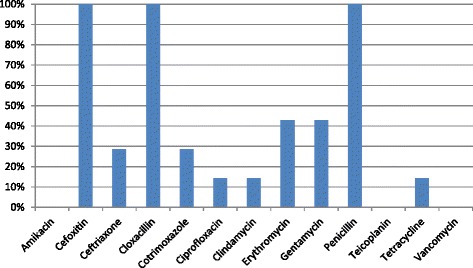
Antibiotic resistance pattern of MRSA

Among 32 *S. aureus* isolates, 7 (21.9 %) were MRSA as detected by resistance to cefoxitin. Resistance to penicillin was 71.9 % whereas all the isolates were sensitive to vancomycin and amikacin (Table 3). Among MRSA isolates 42.9 % (3/7) were resistant to erythromycin and gentamycin. Also all MRSA isolates were sensitive to amikacin, teicoplanin and vancomycin (Fig. 2). Of the 11 erythromycin resistant isolates, erythromycin inducible clindamycin resistance (iMLS_B_) was seen in 45.5 % (5/11) isolates. Among MRSA isolates iMLS_B_ phenotype was observed in 66.7 % (2/3) of erythromycin resistant isolates.

**Table 3:** Antibiotic susceptibility pattern of *S. aureus* isolates (*n* = 32)

Antibiotics	Sensitive (%)	Intermediate (%)	Resistant (%)
Amikacin	100	0	0
Cefoxitin	78.1	0	21.9
Ceftriaxone	68.8	25	6.3
Cloxacillin	68.8	0	31.3
Cotrimoxazole	71.9	0	28.1
Ciprofloxacin	78.1	18.8	3.1
Clindamycin	93.8	0	6.3
Erythromycin	53.1	12.5	34.4
Gentamycin	81.3	3.1	15.6
Penicillin	28.1	0	71.9
Teicoplanin	81.3	9.4	9.4
Tetracycline	93.8	0	6.3
Vancomycin	100	0	0

## Discussion

This study detected a nasal carriage rate of *S. aureus* to be 15.7 % among HCWs which is lower than that reported by studies from elsewhere in Nepal; 20.37–43.80 % [6–9]. This is also lower than that reported (19.80–48 %) internationally [12–18]. However, Khalili et al. have reported a lower nasal carriage rate of 12.67 % [19]. The carriage rate of MRSA was 3.4 % in the present study. Nasal carriage rate of MRSA higher (10 %) [7] and lower (2.32 %) [9] than this study have been previously reported from Nepal. MRSA carriage rate in present study is also lower than internationally reported range (5.8 to 17.8 %) [12, 14, 20–22]. These differences can be attributed to variations in microbiological methods (sampling technique, culture and method of MRSA identification), local infection control standards and the local prevalence of MRSA. Vonberg et al. indicated that screening of HCWs should be performed before starting work duties in order to prevent the detection of transient, short-term MRSA carriage that may occur during a work shift [4]. This may also be a possible cause of low MRSA prevalence in this study. On the other hand, MRSA carriage rate among HCWs is higher in this study as compared to the studies among US population (1.5 %) [23] and that among community adults in the other countries (0.8 to 3.0 %) [24–26] indicating the possibility of hospital acquired MRSA colonization among HCWs.

*S. aureus* carriage rate was highest among doctors (20.8 %) whereas MRSA carriage rate was highest among nurses (7.8 %) in this study. Similar results have been reported by Shibabaw et al. [27]. High risk of colonization with MRSA strains among nurses may be due to their frequent patient contact. HCWs from surgical ward and operating room accounted for 28.6 % of the MRSA carriers each. Similar findings were reported from others studies [28, 29]. This could be due to the traumatic and postoperative immunological suppression of the patients [29]. The nasal carriage of MRSA among HCWs has indicated the chances of transmission of the organism to patients during patient-care. As most isolates belonged to HCWs from surgical ward and operating room, the vulnerability of surgical wound infection with MRSA among the patients, following transmission from the HCWs, further complicating the treatment and recovery, cannot be ignored. Detection of MRSA among HWCs at emergency ward possibly indicates incursion of CA-MRSA into hospital setting, however this has to be confirmed by typing of the strain. The emergency department is a site of high healthcare worker–patient contact, high patient turnover, potentially substantial crowding, and many infected patient wounds that are being drained, explored, and dressed; perhaps it is these characteristics of the emergency department, along with the emergence of CA-MRSA infections in this setting, that explain the colonization observed among HCW in emergency department [30].

The susceptibility testing of MRSA isolates revealed high resistance towards gentamycin and erythromycin (42.9 % each). Low resistance towards ciprofloxacin (14.3 %) and cotrimoxazole (28.6 %) indicates these antibiotics might be an option for empirical therapy of MRSA infections at our hospital. Clindamycin resistance (14.3 %) was also low; however, iMLS_B_ phenotype was seen in 66.7 % of erythromycin resistant MRSA isolates. Though lower resistance of isolates to clindamycin suggests it can be considered for empirical therapy, testing for the detection of inducible clindamycin resistance should be routinely performed in view of high iMLS_B_ phenotype detected among MRSA isolates.

## Conclusion

This study revealed that the prevalence of nasal carriage of *S. aureus* and MRSA among HCWs was low compared to other studies in our country and internationally. The carriage rate of *S. aureus* and MRSA is highest among doctors and nurses respectively. The MRSA carriage rate is high among the HCWs of surgical ward and operating room at our hospital. Inducible clindamycin resistance is high among MRSA isolates. Nasal carriage of *S. aureus* and MRSA among HCWs necessitates the need of control in the frequency of their exposure with the vulnerable patients. The basic infection control measures, screening program and treatment of MRSA-positive HCWs can help as an effective measure to control MRSA infections.

## Abbreviations

MRSA: Methicillin resistant *Staphylococcus aurues*
CA-MRSA: Community acquired methicillin resistant *Staphylococcus aurues*
HA-MRSA: Hospital acquired methicillin resistant *Staphylococcus aurues*
HCWs: Healthcare workers
NICU: Neonatal intensive care unit

## References

[1] Salmenlinna S, Lyytikainen O, Vuopio-Varkila J (2002). Community acquired methicillin-resistant *Staphylococcus aureus*, Finland. Emerg Infect Dis.

[2] Popovich KJ, Weinstein RA, Hota B (2008). Are community-associated methicillin-resistant *Staphylococcus aureus* (MRSA) strains replacing traditional nosocomial MRSA strains?. Clin Infect Dis.

[3] Albrich WC, Harbarth S (2008). Health-care workers: source, vector, or victim of MRSA?. Lancet Infect Dis.

[4] Vonberg RP, Stamm-Balderjahn S, Hansen S, Zuschneid I, Ruden H, Behnke M (2006). How often do asymptomatic healthcare workers cause methicillin-resistant *Staphylococcus aureus* outbreaks? A systematic evaluation. Infect Control Hosp Epidemiol.

[5] Goyal R, Das S, Mathur M (2002). Colonisation of methicillin resistant *S. aureus* among health care workers in a tertiary care hospital of Delhi. Indian J Med Sci.

[6] Pant J, Rai SK (2007). Occurrence of *Staphylococcus aureus* in hospital environment and staffs in teaching hospital in Kathmandu, Nepal. J NAMLS.

[7] Shakya B, Shrestha S, Mitra T (2010). Nasal carriage rate of methicillin resistant *Staphylococcus aureus* among at National Medical College Teaching Hospital, Birgunj, Nepal. Nepal Med Coll J.

[8] Sah P, Rijal KR, Shakya B, Tiwari BR, Ghimire P (2013). Nasal Carriage Rate of Staphylococcus aureus in hospital personnel of National Medical College and Teaching Hospital and their susceptibility pattern. J Health Appl Sci.

[9] Shrestha B, Pokhrel BM, Mohapatra TM (2009). *Staphylococcus aureus* nasal carriage among health care workers in a Nepal hospital. Brazilian J Infect Dis.

[10] Cheesbrough M (2006). District laboratory practice in tropical countries.

[11] CLSI (2007). Performance standards for antimicrobial susceptibility testing. CLSI approved standard M100-S17.

[12] Na’was T, Fakhoury J (1991). Nasal carriage of methicillin-resistant *Staphylococcus aureus* by hospital staff in north Jordan. J Hosp Infect.

[13] Onyemelukwe N, Gugnani HC, Akujieze C (1992). Nasal carriage of *Staphylococcus aureus* in hospital staff and its antibiotic sensitivity in Enugu, Nigeria. J Commun Dis.

[14] Akoua Koffi C, Dje K, Toure R (2004). Nasal carriage of methicillin resistant *Staphylococcus aureus* among health care personnel in Abidjan (Cote d’Ivoire). Dakar Med.

[15] Farzana K, Rashid Z, Akhtar N, Sattar A, Khan JA, Nasir B (2008). Nasal carriage of staphylococci in health care workers: antimicrobial susceptibility profile. Pak J Pharm Sci.

[16] Tejero A, Gutiérrez MA, Aiquel MJ, Brandago M, González C, Broussain MT (1991). Nasal carriage of *Staphylococcus aureus* among personnel working in a teaching hospital. Enferm Infec Microbiol Clin.

[17] Yazgi H, Ertek M, Ozbek A, Kadanali A (2003). Nasal carriage of *Staphylococcus aureus* in hospital personnel and the normal population and antibiotic resistance of the isolates. Mikrobiyol Bul.

[18] Citak S, Bayazit FN, Aksoy F (2011). Nasal carriage and methicillin resistance of *Staphylococcus aureus* in patients and hospital staff in a tertiary referral center setting. Afr J Microbiol Res.

[19] Khalili MB, Sharifi-Yazdi MK, Dargahi H, Sadeghian HA (2009). Nasal colonization rate of *Staphylococcus aureus* strains among health care service employees of teaching university hospitals in Yazd. Acta Med Iran.

[20] Mulqueen J, Cafferty F, Cormican M, Keane JD, Rossney A (2007). Nasal carriage of methicillin-resistant *Staphylococcus aureus* in GPs in the West of Ireland. Br J Gen Pract.

[21] Eveillard M, Martin Y, Hidri N, Boussougant Y, Joly-Guillou ML (2004). Carriage of methicillin-resistant *Staphylococcus aureus* among hospital employees: prevalence, duration, and transmission to households. Infect Control Hosp Epidemiol.

[22] Cesur S, Cokca F (2004). Nasal carriage of methicillin-resistant *Staphylococcus aureus* among hospital staff and outpatients. Infect Control Hosp Epidemiol.

[23] Gorwitz RJ, Kruszon-Moran D, McAllister SK, McQuillan G, McDougal LK, Fosheim GE (2008). Changes in the prevalence of nasal colonization with *Staphylococcus aureus* in the United States, 2001–2004. J Infect Dis.

[24] Abudu L, Blair I, Fraise A, Cheng KK (2001). Methicillin-resistant *Staphylococcus aureus* (MRSA): a community-based prevalence survey. Epidemiol Infect.

[25] Grundman H, Tami A, Hori S, Halwani M, Slack R (2002). Nottingham *Staphylococcus aureus* population study: prevalence of MRSA among elderly people in the community. BMJ.

[26] Jernigan JA, Pullen AL, Partin C, Jarvis WR (2003). Prevalence of and risk factors for colonization with methicillin-resistant *Staphylococcus aureus* in an outpatient clinic population. Infect Control Hosp Epidemiol.

[27] Shibabaw A, Abebe T, Mihret A (2013). Nasal carriage rate of methicillin resistant *Staphylococcus aureus* among Dessie Referral Hospital Health Care Workers; Dessie, Northeast Ethiopia. Antimicrobial Resist Infect Control.

[28] Askarian M, Zeinalzadeh A, Japoni A, Alborzi A, Memish ZA (2009). Prevalence of nasal carriage of methicillin-resistant Staphylococcus aureus and its antibiotic susceptibility pattern in healthcare workers at Namazi Hospital, Shiraz, Iran. Int J Infect Dis.

[29] AL-Talib H, Yean CY, Hasan H, Nik zuraina NMN, Ravichandran M (2013). Methicillin-resistant *Staphylococcus aureus* nasal carriage among patientsand healthcare workers in a hospital in Kelantan, Malaysia. Pol J Microbiol.

[30] Popovich KJ (2010). Commentary*:* the emergency department—an evolving epicenter for healthcare worker acquisition of methicillin‐resistant *Staphylococcus aureus?*. Infect Control Hosp Epidemiol.

